# Industrial-scale separation of high-purity single-chirality single-wall carbon nanotubes for biological imaging

**DOI:** 10.1038/ncomms12056

**Published:** 2016-06-28

**Authors:** Yohei Yomogida, Takeshi Tanaka, Minfang Zhang, Masako Yudasaka, Xiaojun Wei, Hiromichi Kataura

**Affiliations:** 1 Nanomaterials Research Institute, National Institute of Advanced Industrial Science and Technology (AIST), Higashi 1-1-1, Tsukuba, Ibaraki 305-8565, Japan; 2 CNT-Application Research Center, National Institute of Advanced Industrial Science and Technology (AIST), Higashi 1-1-1, Tsukuba, Ibaraki 305-8565, Japan

## Abstract

Single-chirality, single-wall carbon nanotubes are desired due to their inherent physical properties and performance characteristics. Here, we demonstrate a chromatographic separation method based on a newly discovered chirality-selective affinity between carbon nanotubes and a gel containing a mixture of the surfactants. In this system, two different selectivities are found: chiral-angle selectivity and diameter selectivity. Since the chirality of nanotubes is determined by the chiral angle and diameter, combining these independent selectivities leads to high-resolution single-chirality separation with milligram-scale throughput and high purity. Furthermore, we present efficient vascular imaging of mice using separated single-chirality (9,4) nanotubes. Due to efficient absorption and emission, blood vessels can be recognized even with the use of ∼100-fold lower injected dose than the reported value for pristine nanotubes. Thus, 1 day of separation provides material for up to 15,000 imaging experiments, which is acceptable for industrial use.

## Abstract (web summary)

Single-chirality, single-wall carbon nanotubes are attractive for bioimaging applications, however large-scale separation remains a challenge. Here, the authors utilize mixed surfactants for high-resolution single-chirality separation of (9,4) SWCNTs, demonstrating their improved performance for bioimaging.

## Graphical abstract

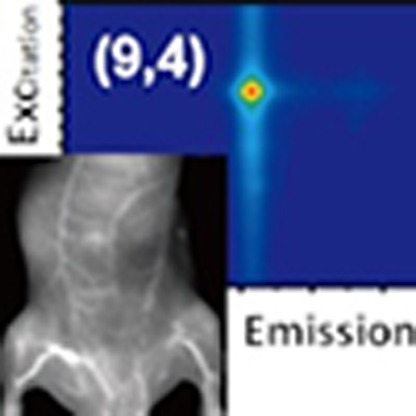

The unique electronic and optical properties of single-chirality, single-wall carbon nanotubes (SWCNTs) have shown promise for developing next-generation of electronic, optoelectronic and biological applications^1^,^2^,^3^. However, even after selective synthesis methods were established^4^,^5^, a mixture of various chirality species is commonly used despite the significant chance for improving the performance of the existing techniques. For instance, a biological imaging method using the near-infrared (NIR) fluorescence of SWCNTs has attracted attention for developing new therapies due to their emission (S_11_) and excitation (S_22_) within the biological transparency window (700–1,400 nm)^6^,^7^,^8^,^9^. However, the SWCNTs were a mixture containing several chirality species with different S_22_ wavelengths, and thus resulted in limited excitation for a light source with single-excitation wavelength. Therefore, the use of single-chirality SWCNTs was desired to improve efficiency. Of the chirality species defined by the chiral indices (*n,m*), (9,4) SWCNTs have a well-designed electronic structure for biological imaging; S_22_ (724 nm)^10^ is in the low-absorption region of oxygenated haemoglobin^11^, and S_11_ (1,101 nm)^12^ is in the low-absorption regions of water^11^, skin^13^ and mucous^13^. In addition, due to the absence of optical absorption of (9,4) SWCNTs in the visible range, transparent flexible transistors are also suitable candidate for the application of these species. Outside of (9,4) SWCNTs, the use of a suitable single species for the application will be promising for the improvement of performance. However, the number of reports is limited, and the number of chirality species used is also limited to a few, such as (6,5)^1^,^2^. Because the electronic and optical properties depend on the chirality of the SWCNTs, it is important to utilize other species for expanding applications.

Various chirality separation methods have been developed to date, including density gradient ultracentrifugation (DGU)^14^, DNA-based ion exchange chromatography (DNA)^15^,^16^ and aqueous two-phase extraction (ATP)^17^,^18^,^19^. However, most methods still have difficulties in achieving high-throughput separation of single-chirality SWCNTs, due to high cost (DNA), limited scalability (DGU), and time-inefficient multiple-step processes (DNA, DGU and ATP). Previously, we developed a gel chromatography method to separate several single-chirality species from high-pressure catalytic CO (HiPco) SWCNTs. Despite the advantages of low cost and high scalability^20^,^21^, this method also faced technical challenges in achieving high purity and throughput. Although the purities of (6,5) and (7,6) were greater than 90% (refs 20, 21), that of (9,4) remained at 46% (ref. 20). The throughput was also suppressed by the repeated chromatographic processes needed for obtaining single-chirality SWCNTs^20^. Basically, this method uses the chirality-selective affinity between sodium dodecyl sulfate (SDS)-wrapped SWCNTs and a dextran-based gel, which is tuned by several parameters: the amount of loaded SWCNTs, the SDS concentration and the temperature. With the opportunity to obtain high-resolution selectivity by introducing new parameters, the purity can be improved even for single-step chromatography. In addition, the previous method used selectivity in the adsorption process only, and an improvement of desorption process would considerably increase the efficiency of chirality separation.

In this study, we develop a new chirality separation method based on the newly discovered chirality selectivity between surfactant-wrapped SWCNTs and the gel. The selectivity is improved by the introduction of a mixed-surfactant system composed of SDS, sodium cholate (SC) and sodium deoxycholate (DOC). In this system, two different selectivities are found; chiral-angle selectivity in SC/SDS system and diameter selectivity in SC/SDS/DOC system. Since the chirality of SWCNTs is determined by the chiral angle and diameter, we demonstrate high-resolution single-chirality separation by combining these independent selectivities in the adsorption and desorption processes. High scalability of this method leads to industrial-scale separation of high-purity (9,4) SWCNTs, which provide improved performance in biological imaging.

## Results

### Chirality selectivity in a mixed-surfactant system

To clarify the effects of the mixed-surfactant system in gel chromatography, chirality selectivity between the surfactant-wrapped SWCNTs and the gel was investigated for two mixed-surfactant systems: SC/SDS and SC/SDS/DOC. HiPco SWCNTs (1.0±0.3 nm) were dispersed in an aqueous solution of 0.5% SC and 0.5% SDS. The SWCNT dispersion was loaded into a column filled with a dextran-based gel (Sephacryl S-200, GE Healthcare). After the elution of unbound metallic SWCNTs, adsorbed SWCNTs were eluted through a stepwise increase in surfactant concentration. For the SC/SDS system, the SDS concentration was increased in a stepwise manner with a fixed concentration of 0.5% SC. For the SC/SDS/DOC system, the DOC concentration was increased in a stepwise manner with fixed concentrations of 0.5% SC and 0.5% SDS. The chirality distribution of the eluted SWCNTs was characterized using optical absorption and photoluminescence spectroscopy.

**Figure 1:**
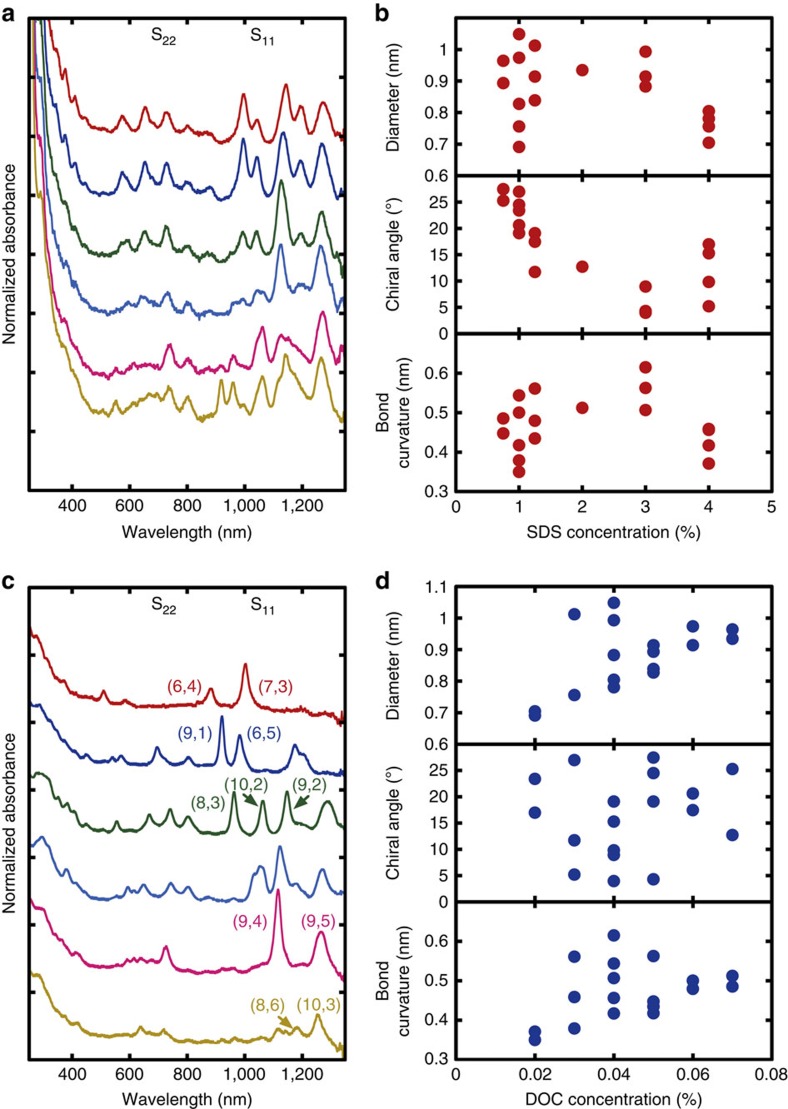
Chirality separation in a mixed-surfactant system. (**a**,**c**) Optical absorption spectra of the eluted SWCNTs at different SDS concentrations in the SC/SDS system (**a**) and at different DOC concentrations in the SC/SDS/DOC system (**c**). These spectra correspond to following surfactant concentrations in order from top to bottom. (**a**) SDS concentrations of 0.75, 1.00, 1.25, 2.00, 3.00 and 4.00%. (**c**) DOC concentrations of 0.02 0.03, 0.04, 0.05, 0.06 and 0.07%. These spectra are normalized at 280 nm and vertically shifted for comparison. (**b**,**d**) The relationship between the elution order of chirality species and their physical properties, including diameter (top of panel), chiral angle (middle of panel) and smallest bond curvature radius (bottom of panel), in the SC/SDS system (**b**) and the SC/SDS/DOC system (**d**). The surfactant concentration for each chirality species was estimated from that of the fraction which shows the brightest Photoluminescence peak for each species. The detailed order of each (*n,m*) species in each system is shown in Supplementary Fig. 3.

Figure 1a shows the optical absorption spectra of the eluted SWCNTs at different SDS concentrations in the SC/SDS system. The absorption spectrum shows a gradual but clear change in shape with increasing SDS concentration, indicating the presence of definite chirality selection in the SC/SDS system. The photoluminescence map more clearly shows that large chiral-angle species including (7,6) and (8,6) were eluted at low-SDS concentrations (0.75% SDS, Supplementary Fig. 1) and that small chiral-angle species including (7,3), (9,1), (8,3) and (9,2) were eluted at high-SDS concentrations (4.0% SDS, Supplementary Fig. 2). Figure 1b shows the relation between elution order of the chirality species (each (*n,m*) species) and their structural characteristics, such as diameter (*D*), chiral angle (*θ*) and smallest bond curvature radius (*R*, 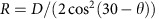) (see also Supplementary Fig. 3). The elution order shows better correlation with the chiral angles than with the other parameters for SDS concentrations lower than 3.0%. When the product of the chiral angle and the bond curvature was plotted against the SDS concentration, the correlation became slightly better (Supplementary Fig. 4). The elution order in the SC/SDS system may have a stronger dependence on the chiral angle at low-SDS concentrations and on the bond curvature at high-SDS concentrations. Thus, the distribution of the eluted SWCNTs changed from more of the species with a larger chiral angle to that with a smaller bond curvature as the SDS concentration increased. This observation of bond curvature selectivity at high-SDS concentration is partially consistent with our previous observation in a single-surfactant system containing SDS^20^, but the chiral-angle selectivity at low-SDS concentration was not observed in the previous work. This selectivity is probably due to the chiral-angle-selective adsorption of SC molecules on SWCNTs, as discussed later.

Figure 1c shows the optical absorption spectra of the eluted SWCNTs at different DOC concentrations in the SC/SDS/DOC system. The result indicates that the eluted SWCNTs change as their S_11_ peaks shift to a longer wavelength with increasing DOC concentration. This finding suggests diameter selectivity because the S_11_ absorption wavelength depends mainly on the diameter^22^. The elution order plotted against DOC concentration shows better correlation with diameter than with the other properties (Fig. 1d). However, the species with diameters >1.0 nm exhibit poor dependence on DOC concentration, as in the cases of (12,1), (11,3) and (10,5). It indicates that the diameter selectivity is limited for small-diameter species. Although this diameter-selective elution is qualitatively consistent with our previous observation using DOC as an eluting solution^23^, the present mixed-surfactant system provides much higher resolution in diameter selection.

These two selectivities in the mixed-surfactant system are completely different from that of the previous SDS, single-surfactant system^20^. It is understood that the chirality selection in the SDS system is based on the bond curvature radius, which arises from the electrostatic interaction between the negative charges of SDS and the positive charges of the oxidized SWCNTs^24^. The larger bond curvature species are susceptible to oxidation and have higher SDS coating coverage, resulting in a suppressed hydrophobic interaction with the gel. In contrast, the selectivity by the introduction of SC and DOC is based on the chiral angle and diameter, respectively, suggesting that each surfactant itself or a mixture with SDS has different interactions from those of the single surfactant of SDS. For SC, it was discussed that SC may have a higher affinity for larger chiral-angle species^25^. Large chiral-angle species have higher SC coverage, and thus suppressed interaction with the gel in this stage of the adsorption process. Therefore, they are eluted at lower surfactant concentrations. In contrast, smaller chiral-angle species are less affected by the SC wrapping and follow the bond curvature dependence usually observed in the SDS, single-surfactant system. For DOC, it was suggested that DOC interacts more strongly with smaller diameter species^23^,^26^. Smaller diameter species are preferentially covered with more DOC and eluted at lower DOC concentrations. For further understanding of their mechanism, however, it is necessary to investigate the contributions of the micelles that form in the mixed-surfactant systems.

### Milligram-scale separation of single-chirality SWCNTs

Since the chirality of SWCNTs is determined by the chiral angle and diameter, it is possible to aim for single-chirality separation using a combination of the chiral angle and diameter selectivities. In this study, (9,4) SWCNTs were selected as a target species for biological imaging. (9,4) SWCNTs have a similar trend to (9,5) SWCNTs in the SC/SDS/DOC system (Supplementary Fig. 3) and thus single-chirality separation of (9,4) SWCNTs is difficult using the system alone. On the other hand, these competitive species have different trend in the SC/SDS system (Supplementary Fig. 3) and thus can be separated using a combination of the two systems. The strategy is as follows. In this study, we used the SC/SDS system for the adsorption process and the SC/SDS/DOC system for the desorption process. This method can divide such competitive species into adsorbed species containing the targeted one and unbound species containing the competitor in the first chiral-angle-selective adsorption process. The targeted species can be eluted by the subsequent diameter-selective desorption process. The adsorption process was performed in a solution of 0.5% SC and 1.0% SDS, which allow chiral-angle-selective adsorption of (9,4) SWCNTs excluding (9,5) SWCNTs. To aim for practical use, a liquid chromatography system was used. First, separation conditions were optimized for a small-scale separation using 1.0 ml of the SWCNT dispersion and the column with 4.7 ml of gel. Next, the scalability of separation was investigated for a large-scale separation using 80–400 ml of the SWCNT dispersion and the column with 430 ml of gel.

**Figure 2:**
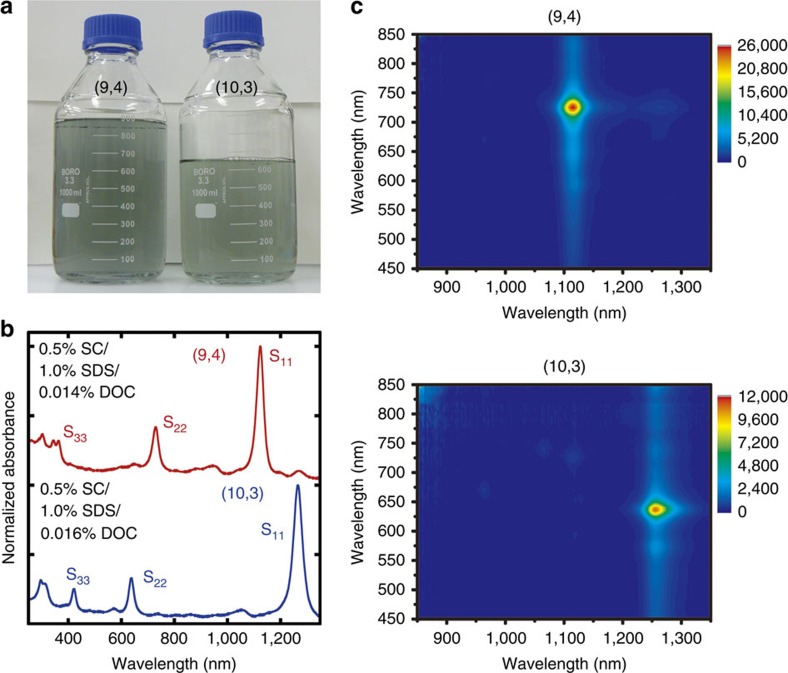
High-throughput, single-chirality separation. (**a**) Photographs of the separated (9,4) SWCNT dispersion (900 ml, left of panel) and (10,3) SWCNT dispersion (600 ml, right of panel). (**b**) Optical absorption spectra of the separated (9,4) (top of panel) and (10,3) SWCNTs (bottom of panel). The spectra are normalized at the S_11_ peaks and shifted vertically for comparison. (**c**) Photoluminescence maps of the separated (9,4) (top of panel) and (10,3) SWCNTs (bottom of panel).

For the small-scale separation (Supplementary Fig. 5), the resulting spectra show the successful separation of single-chirality species, including (9,4) and (10,3) elution at 0.14 and 0.16% DOC, respectively. Similar elution behaviour was observed, even in the large-scale separation. Figure 2a–c shows a photograph of the sample bottles, optical absorption spectra and the photoluminescence maps of each SWCNT fraction obtained from the large-scale separation. Despite the ∼100-fold scale-up, the separation conditions and quality are comparable to those obtained from the small-scale separation, which indicates the high scalability of this method. The S_11_, S_22_ and S_33_ peaks are clearly visible for each single-chirality SWCNT fraction, suggesting that high-purity single-chirality SWCNTs were obtained. Compared with those in Fig. 1c, the purities of (9,4) and (10,3) are highly improved, because of the absence of competitors, such as (9,5) for (9,4), and (8,6) for (10,3). The reproducibility of the separation was confirmed for >10 times using the same gel column, which reduces the cost per one separation. From a simple estimation, the cost of the gel is lower than one-thirds of the cost for used pristine HiPco SWCNTs per separation.

To evaluate this method in comparison with others, the purities, production yields and throughputs of (9,4) and (10,3) were estimated from the optical absorption spectra. Table 1 shows the results. The estimated purities of single-chirality SWCNTs were 93% for (9,4) and 96% for (10,3), which are the highest among the reported values^15^,^20^. The production yields of these high-purity, single-chirality SWCNTs were also estimated to be the highest values^15^. The yield is a trade-off with the purity and increases when lower purity fractions are included. Here, only fractions with purities >90% were counted. Owing to the high yield and fast separation, the 1-day throughput of single-chirality SWCNTs reached the milligram-scale, 1.2 mg per day. Furthermore, increasing the amount of SWCNT dispersion easily enhanced the throughput. DOC was initially added to the SWCNT dispersions to be 0.13%, and the adsorption step was performed in a solution of 1.0% SDS, 0.5% SC and 0.13% DOC, where only (9,4) and (10,3) SWCNTs can be adsorbed. Because of the effective use of adsorption sites for (9,4) and (10,3) SWCNTs, the amount of adsorbed (9,4) and (10,3) SWCNTs was enhanced, resulting in an enhanced throughput of up to 5.2 mg per day in the case of a fivefold increase of loaded SWCNTs. We believe this throughput can be acceptable for industrial-scale use. Since 100-fold scalability was confirmed in this method, we can easily apply this method to larger system, possibly resulting in further enhanced throughput as needed. However, there are several practical issues. This throughput does not include the time necessary for the preparation of the SWCNT dispersion. It is important to develop an efficient process for preparing the SWCNT dispersion for practical separation. Another issue is the total yield of single-chirality SWCNTs from the starting material, which can be enhanced by the separation of other species. Although this study focused on (9,4) SWCNTs, the capability to separate 5 additional single-chirality species, (6,4), (7,3), (6,5), (10,2) and (8,6), was confirmed for various surfactant compositions (Supplementary Fig. 6). Optimizing the combination of two selectivities will allow many more species to be separated, leading to an enhanced total yield. Our productivity cannot be directly compared with various other existing methods because their actual productivities have not been reported. However, we note that this method, where the chiral angle and diameter selectivities were combined, is a different concept, and this report is a clear demonstration with evidence of a high-throughput separation of high-purity single-chirality SWCNTs.

### Biocompatible single-chirality (9,4) SWCNTs

**Figure 3:**
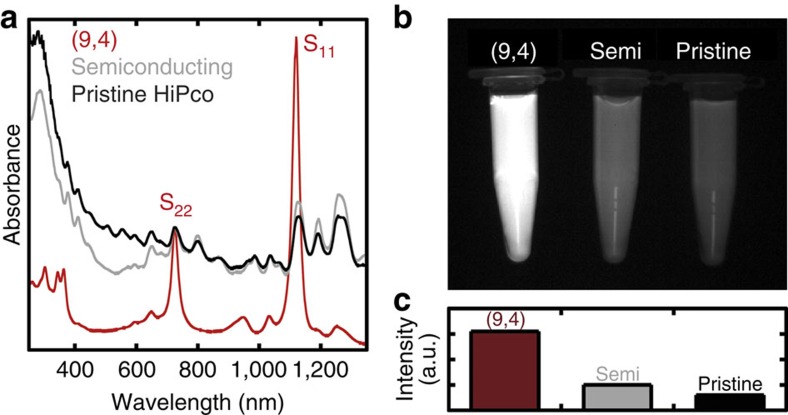
Optical characteristics of biocompatible SWCNTs. For all optical measurements, diluted samples (× 40) were used. (**a**) Optical absorption spectra of biocompatible-surfactant-coated (9,4) SWCNTs (red), semiconducting SWCNTs (grey) and pristine HiPco SWCNTs (black). The concentration of each sample was adjusted to obtain the same absorbance at ∼725 nm, corresponding to S_22_ of (9,4) SWCNTs. The resulting mass concentrations of (9,4), semiconducting, and pristine HiPco SWCNTs were 0.17, 0.52 and 0.64 mg ml^−1^, respectively. (**b**) NIR fluorescence image of the samples. (**c**) NIR fluorescence intensity of the samples.

To use the separated (9,4) SWCNTs for biological imaging, the surfactant on the SWCNTs was replaced with a biocompatible surfactant, 1,2-distearoyl-sn-glycero-3-phosphoethanolamine-N-[amino(poly(ethylene-glycol))-5000] (DSPE-PEG)^8^. For comparison, reference samples including semiconducting (s-SWCNTs) and pristine HiPco SWCNTs (p-SWCNTs) were also prepared in the same manner. Figure 3a shows the optical absorption spectrum of DSPE-PEG-coated SWCNTs. In this study, 735-nm light-emitting diode (LED) was used as the excitation source, which strongly overlaps with S_22_ of (9,4) SWCNTs (725 nm) (Supplementary Fig. 7). To make the excitation intensity of each sample as equal as possible, their mass concentration was adjusted to make their S_22_ peaks at 725 nm equivalent, which is the nearest to LED wavelength of 735 nm. Final mass concentrations were estimated to be 0.17 mg ml^−1^ for (9,4) SWCNTs, 0.52 mg ml^−1^ for s-SWCNTs and 0.64 mg ml^−1^ for p-SWCNTs. According to the absorption per unit mass estimated from the mass concentration, (9,4) SWCNTs exhibited enhanced excitation, which was 3.1- and 3.8-times higher than that of s-SWCNTs and p-SWCNTs, respectively.

To evaluate the relative fluorescence brightness, each sample was imaged under excitation at 735 nm (Fig. 3b). Despite the lower mass concentration, (9,4) SWCNTs exhibited 3.1- and 5.0-times higher photoluminescence intensities compared with those of s-SWCNTs and p-SWCNTs, respectively (Fig. 3c). Because of the equivalent absorption at the excitation wavelength, these values are ideally identical to experimental photoluminescence efficiencies. Given the absorption and photoluminescence intensity per unit mass, it was estimated that (9,4) SWCNTs are 19-times brighter than p-SWCNTs at the same mass concentration. The extremely bright fluorescence of (9,4) SWCNTs provides efficient biological imaging. Although it is challenging to understand this high-photoluminescence efficiency of (9,4), high purity of (9,4) SWCNTs should provide some contributions: suppressed reabsorption of S_11_ emission and/or suppressed S_22_ absorption, by a non-luminescent metallic species and/or a semiconducting species such as (11,4) and (14,1) that have longer photoluminescence wavelengths than 1,350 nm, where photons are absorbed by water. Further experiments will be necessary to understand this result more.

### Efficient bioimaging using single-chirality (9,4) SWCNTs

**Figure 4:**
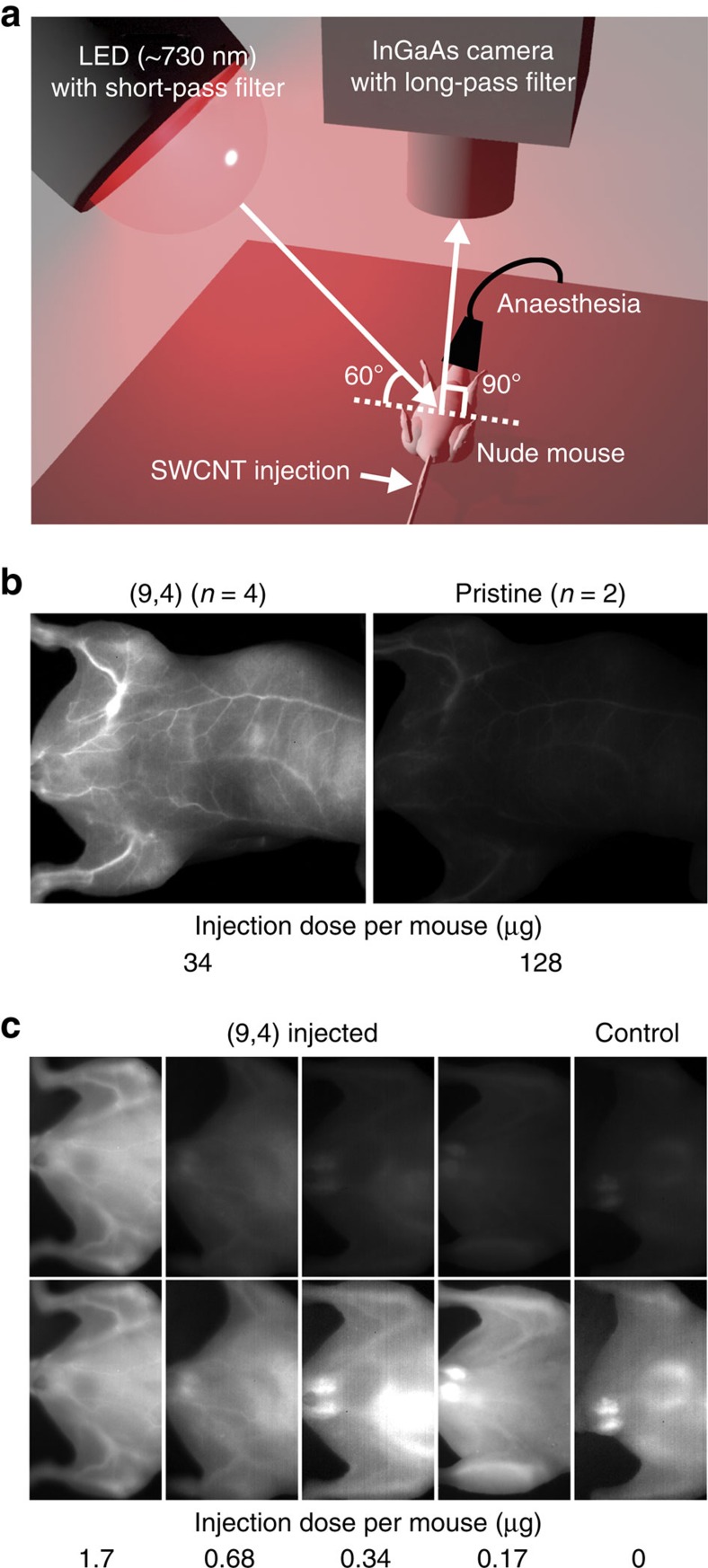
High-efficiency bioimaging using single-chirality (9,4) SWCNTs. (**a**) Schematic representation of the imaging setup. (**b**) NIR fluorescence images of mice injected with (9,4) (left of panel) and pristine HiPco SWCNTs (right of panel) under the same imaging conditions. (**c**) NIR fluorescence images of mice with different injected doses of (9,4) SWCNTs (1.7–0.17 μg per mouse) and a control mouse without injection, under the same imaging conditions (top of panel) and after adjustment for optimal contrast (bottom of panel).

Nude mice (*n*=4) were intravenously injected with the biocompatible (9,4) SWCNTs (100–200 μl, 0.17 mg ml^−1^) and imaged under illumination by the 735-nm LED (Fig. 4a). For comparison, nude mice (*n*=2) injected with p-SWCNTs (100–200 μl, 0.64 mg ml^−1^) were also imaged under the same imaging conditions. Figure 4b shows the whole body of a mouse injected with (9,4) SWCNTs (34 μg per mouse) and p-SWCNTs (128 μg per mouse) under the same imaging conditions. As is the case in the photoluminescence measurement of the dispersion, the (9,4) SWCNTs provided much brighter vascular imaging than did the p-SWCNTs despite the lower injected dose. Although the image of the p-SWCNTs exhibited was similar to that of the (9,4) SWCNTs after an adjustment for the optimal contrast, the (9,4) SWCNTs led to clearer images due to the higher signal-to-noise ratio (Supplementary Fig. 8). This bright fluorescence from (9,4) SWCNTs provides high-temporal imaging resolution of <100 ms. The temporal resolution is an important parameter for inner-organ imaging using principal component analysis, which was used to convert the temporal difference into dynamically enhanced contrast with spatial registration^27^. In addition, (9,4) SWCNTs have another advantage for deeper inner organs due to the low tissue absorption. For instance, a heart and lungs were clearly distinguished immediately after the intravenous injection of (9,4) SWCNTs, followed by the appearance of the liver (Supplementary Fig. 9 and Supplementary Movie 1). Combining (9,4) SWCNTs with this observation system will provide bright organ imaging of any mouse body in any view (Supplementary Fig. 10).

The minimum injected dose was investigated by changing the injected dose of (9,4) SWCNTs. Figure 4c, top shows mice injected with various doses of (9,4) SWCNTs (1.7–0.17 μg per mouse) and a control mouse without an injection under the same imaging conditions. With decreasing injected dose, the NIR fluorescence brightness gradually decreased and became nearly as dark as the low tissue autofluorescence of the control mouse. In contrast, blood vessels were clearly distinguished at the lowest injected dose of 0.17 μg per mouse by adjusting the contrast and brightness of the images (Fig. 4c, bottom). This obtained value is ∼20-fold and 100-fold lower than the reported value for chirality-separated (3 μg per mouse)^8^ and pristine SWCNTs (20 μg per mouse)^6^, respectively. This low dose of (9,4) SWCNTs reduces any possible risk for the subject. Because of the highest throughput of (9,4) SWCNTs (2.7 mg per day) and the injected dose of (9,4) SWCNTs (0.17 μg per mouse), it is possible to obtain samples for 15,000 imaging experiments at the maximum by separation within 1 day. This separation must be industrial scale for biological imaging.

## Discussion

The advantage of (9,4) SWCNTs for biological imaging is not only the efficient absorption and photoluminescence intensity, but also their optimal photoluminescence wavelength for biological imaging. Supplementary Fig. 11 shows the extinction coefficient of mouse skin plotted against wavelength. The obtained spectra show that extinction minimum is in the 1,100 nm region, which corresponds to S_11_ of (9,4) SWCNTs. This finding supports (9,4) SWCNTs as the best candidate for efficient imaging through skin. However, the wavelength dependence of the extinction properties differs depending on the biological tissue. In the case of imaging through a skull, it was reported that extinction minimum is in the 1,300 nm region due to contribution by scattering^9^. Supplementary Fig. 12 shows the absorption coefficient of water and the reduced scattering coefficient of various tissues based on the previously reported scattering properties^13^,^28^,^29^. Generally, the absorption coefficient of most biological tissues has a trend similar to that of water and has a higher extinction in the longer wavelength region. In contrast, the scattering coefficients have the inverse trend, and extinction is more suppressed in the longer wavelength region. Given these factors, there are two suitable candidates depending on the scattering profile of tissues. In the cases of tissues with weak wavelength dependence, such as skin and mucous, the optical absorption should be the dominant factor in extinction, resulting in an advantage in the 1,100 nm region. In the other cases, the scattering should be the dominant factor, leading to an advantage in the 1,300 nm region. In addition, the scattering decreases the imaging contrast. If high resolution is the purpose of accepting a high dose, then the 1,300-nm region should be the best candidate.

In conclusion, we demonstrated high-throughput separation of high-purity, single-chirality SWCNTs using mixed-surfactant gel chromatography. The mixed-surfactant system composed of SDS, SC and DOC provided chirality selectivity between surfactant-wrapped SWCNTs and the gel: chiral-angle selectivity in the SC/SDS system and diameter selectivity in the SC/SDS/DOC system. By combining chiral-angle selectivity for adsorption and diameter selectivity for desorption, we developed a method for efficient single-chirality separation, resulting in the successful separation of 7 single species. This simple and fast process enabled the milligram-scale separation of single-chirality (9,4) and (10,3) SWCNTs with high purity (>90%), high yield and the high throughput of 5.2 mg per day. Since 100-fold scalability was confirmed in this method, we can apply it to larger systems, which may lead to enhanced throughput as needed. In addition, NIR fluorescence imaging of mouse vasculatures was performed using separated (9,4) SWCNTs as the fluorescence probe. Due to the high absorption and emission intensity per unit mass, the surfactant-exchanged, biocompatible (9,4) SWCNTs exhibited ∼20-fold brighter fluorescence than the pristine HiPco SWCNTs, providing clear vascular and deep inner-organ imaging with a high signal-to-noise ratio and high-temporal resolution. Blood vessels could be recognized even with the use of ∼100-fold lower injected dose than previously reported value for pristine SWCNTs, which help to reduce any possible risk for mice. The advantage of the minimum light loss through the skin and mucous tissues allow further advances in biological imaging. This efficient imaging can be performed 15,000 times at the maximum using the (9,4) SWCNTs from the output of one day of separation, which is acceptable for industrial use. This separation method has many advantages compared with the other reported methods because of its simplicity, low cost, high purity, high yield and high throughput. We believe that this industrial-scale single-chirality separation may be applied to the other single-chirality SWCNTs and could lead to new stages for various applications of SWCNTs.

## Methods

### Dispersion of SWCNTs

SWCNTs produced by high-pressure catalytic CO (HiPco) decomposition (R1831, 1.0±0.3 nm, NanoIntegris, Inc.) with a content percentage of 5.2% were used as the starting material. First, 100 mg of HiPco SWCNTs was dispersed in 100 ml of a 0.5% SC (99%, Sigma-Aldrich) aqueous solution (all surfactant concentrations were weight per cent), which is known to be an efficient dispersant of SWCNTs^30^, using an ultrasonic homogenizer (Sonifier 450D, Branson) equipped with a 0.5-inch flat tip for 3 h at a power density of 30 W cm^−2^. To prevent heating during sonication, the bottle containing the sample solution was immersed in a bath of cold water. To remove the residual catalytic metal particles, nanotube bundles and impurities, the dispersed sample solution was centrifuged at 210,000*g* for 120 min in an angle rotor (S50A, Hitachi Koki). The top 80% of the supernatant was collected and used for gel chromatography and biological imaging as pristine HiPco SWCNTs. Before separation, SDS (99%, Sigma-Aldrich), which is necessary for the separation of SWCNTs^31^, was added to the dispersion according to the separation conditions.

### Chirality separation in mixed-surfactant systems

Approximately 3 ml of allyl dextran-based gel beads (Sephacryl S-200 HR, GE Healthcare) was packed in a plastic medical syringe equipped with a cotton filter on the outlet of the syringe. The adsorption procedure was performed as follows. After equilibration with a 0.5% SDS and 0.5% SC solution, 0.6 ml of the SWCNT dispersion with 0.5% SDS and 0.5% SC was loaded into the column. To elute unbound SWCNTs, the 0.5% SDS and 0.5% SC solution was loaded until no nanotubes were detected in the eluent. The elution procedures were different depending on the system. For the SC/SDS system, the adsorbed SWCNTs were eluted and collected by increasing the SDS concentration (0.75, 1.0, 1.25, 1.5, 2.0, 2.5, 3.0, 4.0 and 5.0%) with a fixed concentration of 0.5% SC. For the SC/SDS/DOC system, the adsorbed SWCNTs were eluted and collected by increasing the concentration of DOC (99%, Sigma-Aldrich) from 0.01 to 0.10% in 0.01% steps with fixed concentrations of 0.5% SC and 0.5% SDS. The chirality distributions of the eluted SWCNTs were determined using optical absorption and photoluminescence spectroscopy.

### Single-chirality separation of (9,4) and (10,3) SWCNTs

A chromatography system (AKTAexplorer 10S, GE Healthcare) was installed in a chamber with a defined temperature at 18–20°C. For the small-scale separation, ∼4.7 ml of allyl dextran-based gel beads (Sephacryl S-200 HR) was packed in a column (Tricorn 10/50, GE Healthcare). After equilibration with a 1.0% SDS and 0.5% SC solution, 1.0 ml of the SWCNT dispersion with 1.0% SDS and 0.5% SC, followed by the 1.0% SDS and 0.5% SC solution, was loaded into the column at a flow rate of 1 ml min^−1^. The adsorbed SWCNTs were eluted and collected by increasing the DOC concentration from 0.01 to 0.2% in 0.01% steps with fixed concentrations of 0.5% SC and 1.0% SDS. For the large-scale separation, ∼430 ml of Sephacryl S-200 gel was packed in a column (HiScale 50/20, GE Healthcare). The adsorption procedure was performed in the same manner as for the small-scale separation. Approximately 80 ml of the SWCNT dispersion was loaded into the column at a flow rate of 10 ml min^−1^. After collecting fractions containing small-diameter SWCNTs at low DOC concentrations, (9,4) and (10,3) SWCNTs were eluted at 0.14 and 0.16% DOC, respectively. This procedure takes ∼6 h for a single cycle of separation. For further enhancement of the throughput, we changed the adsorption conditions and increased the amount of loaded SWCNT dispersion. The adsorption step was performed in a solution of 1.0% SDS, 0.5% SC and 0.13% DOC, and ∼400 ml of the SWCNT dispersion was loaded into the column. (9,4) and (10,3) SWCNTs were eluted at 0.14 and 0.16% DOC, respectively. This procedure takes ∼4.8 h for one cycle. The separation of other single-chirality SWCNTs ((6,4), (7,3), (6,5), (10,2) and (8,6)) is described in detail in the Supplementary Methods 1.

### Optical spectra measurements

The optical absorption spectra of the SWCNTs were measured from 250 to 1,350 nm using a UV-vis-NIR spectrometer (UV-3600, Shimadzu) with a quartz cuvette and a path length of 10 mm. The spectral slit width was 2 nm. The photoluminescence spectra of the SWCNTs were measured as a function of excitation and emission wavelengths using a spectrofluorometer (NanoLog, HORIBA) equipped with a liquid nitrogen-cooled InGaAs near-IR array detector. The excitation wavelength was varied from 450 to 850 nm in 5-nm steps, and the emission wavelength was varied from 850 to 1,350 nm. The spectral slit widths were 7 nm for both excitation and emission. The raw data were corrected using instrumental factors. The optical extinction spectrum of mouse skin was measured from 250 to 1,350 nm using a UV-VIS-NIR spectrometer (UV-3700, Shimadzu) equipped with an integrating sphere. The mouse skin was completely removed from the back of a nude mouse and immediately used for the optical measurements. The thickness of the mouse skin was measured using digital calipers and estimated to be 0.35 mm. The spectral slit width was 8 nm.

### Evaluation of the separated (9,4) and (10,3) SWCNTs

The purity was evaluated using a previously described method^20^. PeakFit software was used to simulate the NIR spectra of the individual (*n,m*) species. The optical absorption spectrum was fitted to the superposition of simulated peaks of the individual (*n,m*) species in the range from 800 to 1,350 nm, and the purity was computed as the ratio of the area of the dominant absorption peak to the sum of all peak areas. The production yield and the throughput were evaluated using the mass concentration, which was estimated from the absorbance in the ultraviolet region (∼280 nm). The absorption spectrum intensity was converted to the SWCNT mass concentration^32^. The production yield was computed as the ratio of the mass of the purified species to the total mass of the initially loaded HiPco SWCNTs. The throughput was the estimated total mass of single-chirality SWCNTs collected within 1 day.

### Preparation of biocompatible (9,4) SWCNTs

The surfactant on the (9,4) SWCNTs was replaced with a biocompatible surfactant, DSPE-PEG (Sunbright)^8^. The separated (9,4) SWCNTs were concentrated by adsorption to the gel again after dilution with the same amount of a 0.5% SC and 1.0% SDS solution. The adsorbed (9,4) SWCNTs were eluted with 0.5% SC solution. Next, 1.0 mg of DSPE-PEG was added to 1.0 ml of the SWCNT dispersion. The dispersion was sonicated in a bath sonicator for 10 min and filtered to remove SC using a centrifugal filter unit (Amicon Ultra-15 with Ultracel-3 membrane, Merck Millipore) for 25 min at 3,000*g*. The concentrate was diluted with water, and this process was repeated more than five times. Before use, the dispersion was centrifuged again for 90 min at 10,000*g* to remove any possible aggregates. For comparison, reference samples including semiconducting SWCNTs separated from HiPco SWCNTs and pristine HiPco SWCNTs were also prepared.

### Mouse handling and injection

Female 6-week-old nude mice (BALB/cAJal-nu/nu) were purchased from CLEA Japan, Inc., and housed for 8 weeks. All animal experiments were performed in accordance with the regulations approved by the Animal Care and Use Committee of AIST. For comparative vascular imaging, solutions of (9,4) and pristine HiPco SWCNTs dispersed by DSPE-PEG were intravenously injected into the tail vein of a mouse using a 29-gauge needle. Each mouse was anaesthetised by inhalation of isoflurane inhalation solution (Pfizer Inc.) and placed on the imaging stage within 5 min of injection. For dynamic imaging, a 29-gauge needle with a cannula (Instech Laboratories, Inc.) was inserted into the tail vein before anaesthetisation. The (9,4) SWCNT dispersion was injected to the anaesthetised mouse after the imaging began. For low-dose imaging, the (9,4) SWCNT dispersion was diluted with a 1% DSPE-PEG solution. All SWCNT dispersions were mixed with 10 × PBS to obtain a final PBS concentration of 1 × before injection. For each mouse, 100 or 200 μl of the SWCNT solution was injected.

### NIR fluorescence imaging

NIR fluorescence images were collected using a home-built imaging system (Fig. 4a). The excitation light was provided by a 48 W, 735 nm LED (IP-307TC5, Lanics) and filtered by an 800-nm short-pass filter (#84-729, Edmund Optics Inc.). The emitted light from the mouse was filtered by a 1,000-nm long-pass filter (ITF-50S-100RM, Sigma Koki) to reject excitation light and autofluorescence, and then collected by a 640 × 512 pixel two-dimensional InGaAs array (NIRvana 640ST, Princeton Instruments) equipped with an objective lens (COSMICAR, PENTAX). The camera was set vertically at 16 cm from the imaging stage. The excitation LED was located at 14 cm from the imaging stage with the elevation angle 60°. LED light intensity at the surface of the mouse was about 40 mW cm^−2^. Temperature raise of the mouse body by LED irradiation was confirmed to satisfy the regulations approved by the Animal Care and Use Committee of AIST. The exposure time for all images was 100 ms. The numerical aperture of the lens was adjusted depending on the experiments: 2.8 and 4 for comparative vascular imaging in 100 and 200 μl injection, respectively, 5.6 for dynamic imaging, and 1.4 for low-dose imaging.

### Data availability

The authors declare that the data supporting the findings of this study are available within the article and its Supplementary Information files.

## Additional information

**How to cite this article:** Yomogida, Y. *et al.* Industrial-scale separation of high-purity single-chirality single-wall carbon nanotubes for biological imaging. *Nat. Commun.* 7:12056 doi: 10.1038/ncomms12056 (2016).

## Supplementary Material

Supplementary Information Supplementary Figures 1-12, Supplementary Methods and Supplementary References.

Supplementary Movie 1 Time-resolved NIR fluorescence images of a mouse injected with (9,4) SWCNTs.

## Figures and Tables

**Table 1:** Evaluated values of separated (*n,m*) species compared with previous studies.

		**Present**	**Previous [20]**	**DNA [15]**
Purity of single chirality (%)	(9,4)	93	46	60
	(10,3)	96	69	—
Yield of single chirality per loaded HiPco (%)	(9,4)	0.80	**—**	0.5
	(10,3)	0.50	**—**	**—**
Throughput of single chirality (mg per day)	Total	1.2	**—**	**—**
