# Supplementary material for: Industrial-scale separation of high-purity single-chirality single-wall carbon nanotubes for biological imaging
Source: Nat Commun. 2016 Jun 28;7:12056. doi: 10.1038/ncomms12056 (PMC4931232; doi:10.1038/ncomms12056)
Supplement: Supplementary Information — Supplementary Figures 1-12, Supplementary Methods and Supplementary References. [file ncomms12056-s1.pdf]

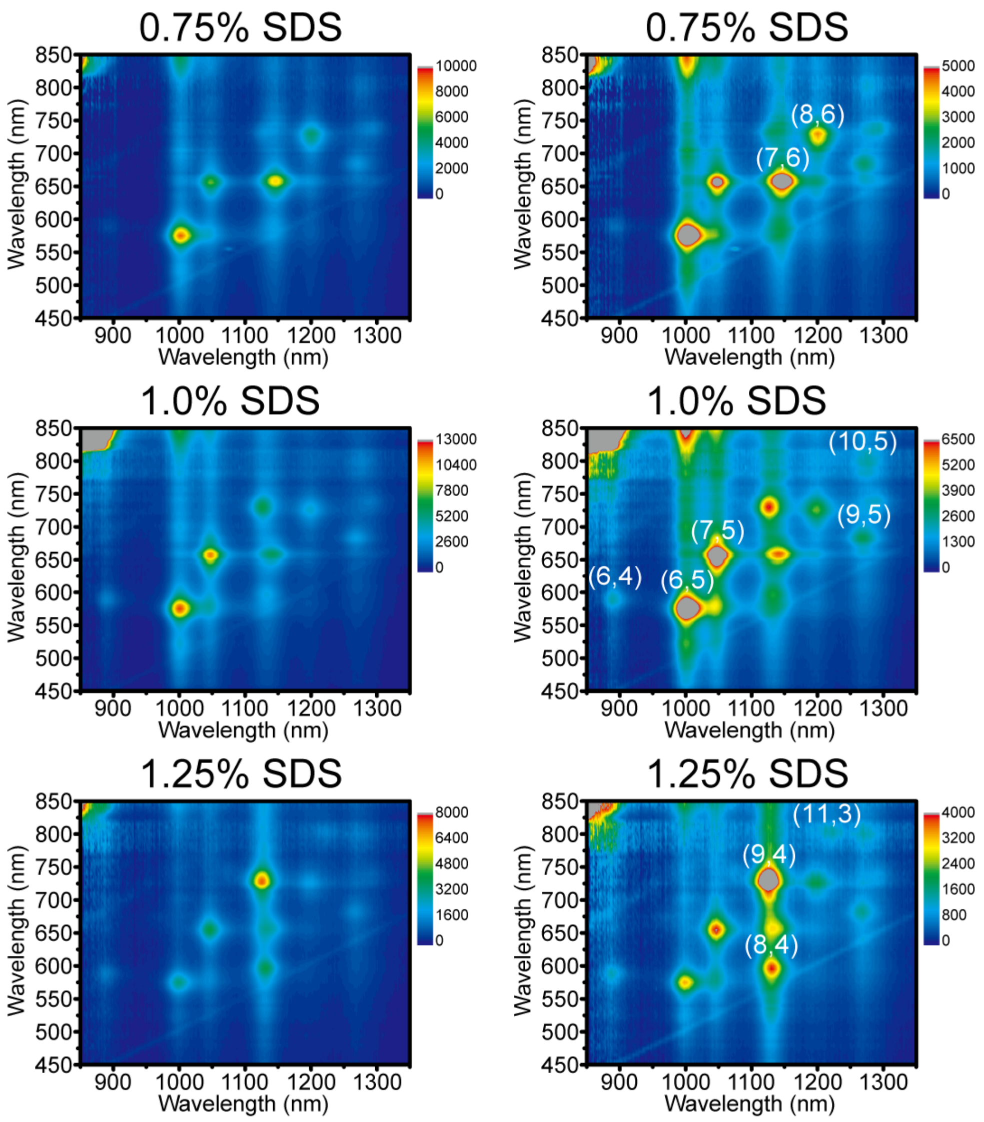

Supplementary Figure 1. PL maps of the eluted SWCNTs at low SDS concentrations in the SC (0.5%) /SDS system

These spectra correspond to SDS concentrations of 0.75 (top of panel), 1.0 (middle of panel), and 1.25% (bottom of panel) and are plotted according to the highest peak (left of panel) and half of the peak (right of panel). The chiral indices show the brightest peak of (*n*,*m*) species among various SDS concentrations in Supplementary Figs. 1 and 2.

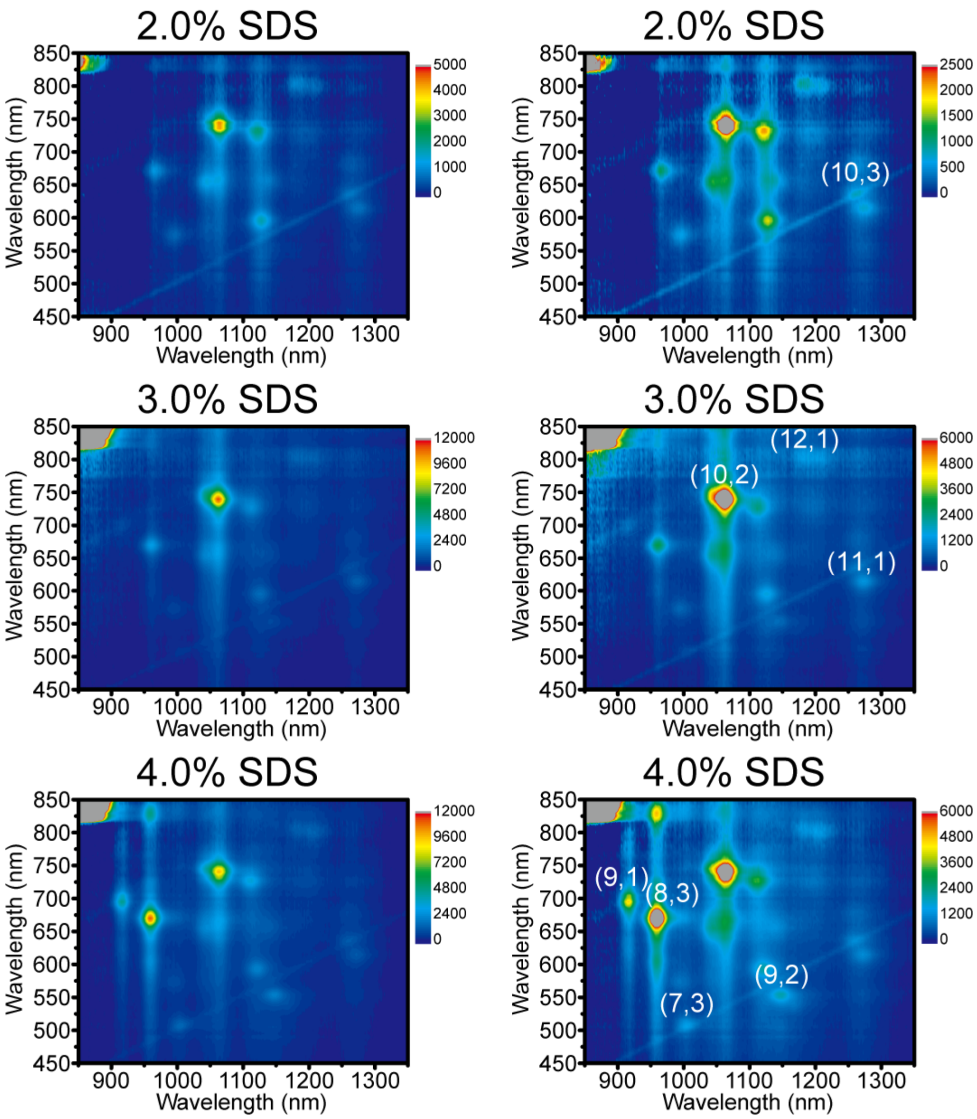

Supplementary Figure 2. PL maps of the eluted SWCNTs at high SDS concentrations in the SC (0.5%) /SDS system

These spectra correspond to SDS concentrations of 2.0 (top of panel), 3.0 (middle of panel), and 4.0% (bottom of panel) and are plotted according to the highest peak (left of panel) and half of the peak (right of panel). The chiral indices show the brightest peak of (*n*,*m*) species among various SDS concentrations in Supplementary Figs. 1 and 2.

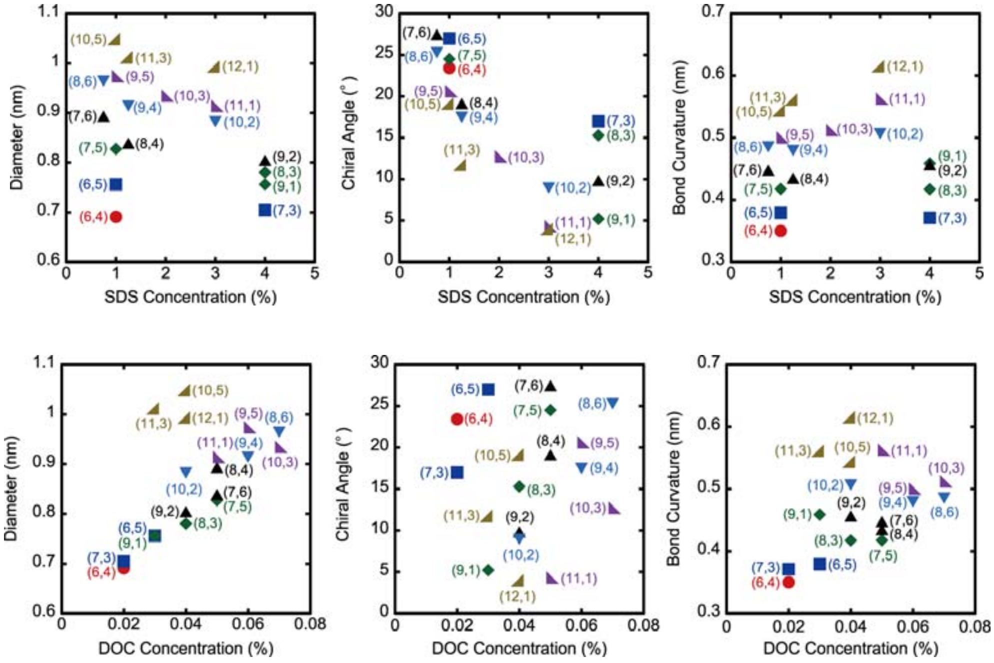

Supplementary Figure 3. Relationship between the elution order of (*n*,*m*) species and structural parameters

Relationship between the elution order of (*n,m*) species and diameter (left of panel), chiral angle (middle of panel), and bond curvature radius (right of panel), in the SC/SDS system (top of panel) and the SC/SDS/DOC system (bottom of panel). The surfactant concentration for each chirality species was estimated from that of the fraction which shows the brightest PL peak for each species. Different symbols indicate different families of SWCNTs ($2n + m$ = 16, 17, 19, 20, 22, 23, and 25).

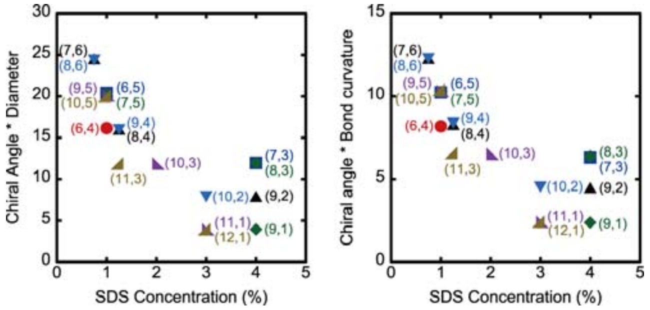

Supplementary Figure 4. Relationship between the elution order of (*n*,*m*) species and structural characteristics

Relationship between the elution order of (*n*,*m*) species and the product of chiral angle and diameter (left of panel), and the product of chiral angle and bond curvature radius (right of panel), in the SC/SDS system. Different symbols indicate different families of SWCNTs ($2n + m$ = 16, 17, 19, 20, 22, 23, and 25).

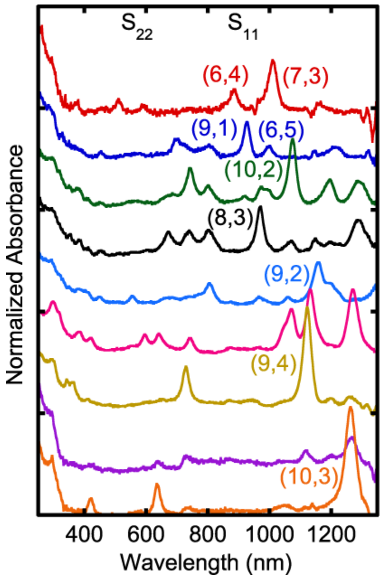

Supplementary Figure 5. Optical absorption spectra of the eluted SWCNTs at different DOC concentrations

These spectra correspond to DOC concentrations of 0.06, 0.07, 0.08, 0.09, 0.10, 0.12, 0.14, 0.15, and 0.16% in order from top to bottom. These spectra are normalized at 280 nm and vertically shifted for comparison.

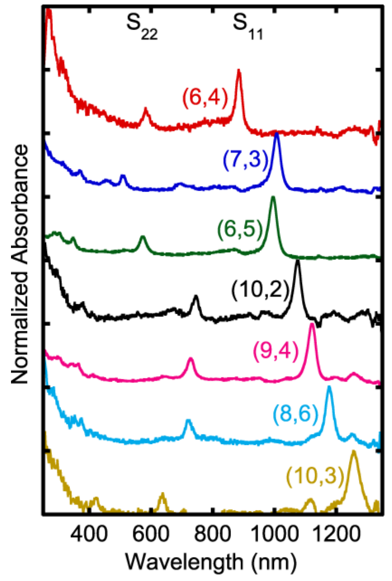

Supplementary Figure 6. Optical absorption spectra of the seven single-chirality SWCNTs

These spectra are normalized at the S_{11} peaks and vertically shifted for comparison.

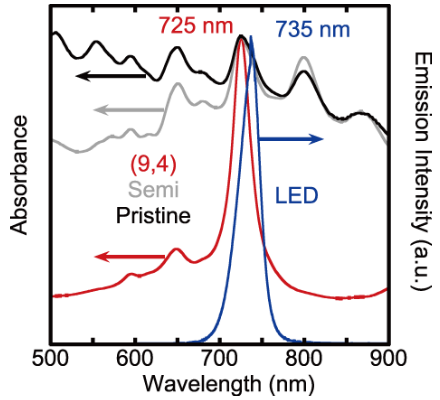

Supplementary Figure 7. Optical absorption spectra of DSPE-PEG-coated SWCNTs and emission spectrum of LED used for excitation

These absorption spectra correspond to (9,4) SWCNTs (red), semiconducting SWCNTs (gray), and pristine HiPco SWCNTs (black). The emission spectrum of LED filtered by a 800-nm short-pass filter (blue) is also shown.

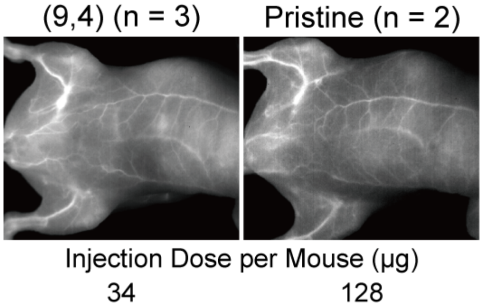

Supplementary Figure 8. NIR fluorescence images of mice after adjustment for optimal contrast of the images

The mice were injected with (9,4) (left of panel) and pristine HiPco SWCNTs (right of panel), which appear in Figure 4b.

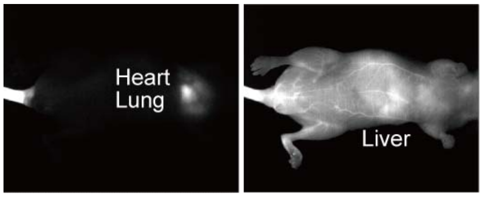

Supplementary Figure 9. Time-resolved NIR fluorescence images of the same mouse injected with (9,4) SWCNTs

These images were collected after 2 (left of panel) and 20 s (right of panel) of injection.

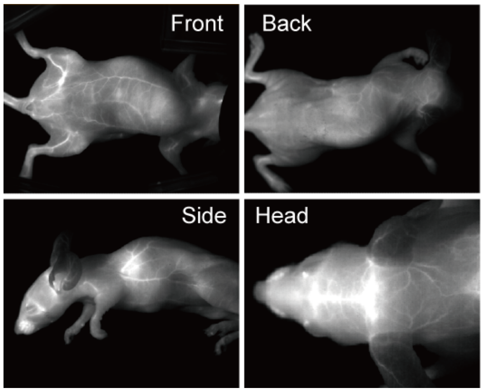

Supplementary Figure 10. NIR fluorescence images of the same mouse injected with (9,4) SWCNTs

These images were viewed from the front (top left of panel), back (top right of panel), and side (bottom left of panel). The magnified image of the mouse head is also shown (bottom right of panel).

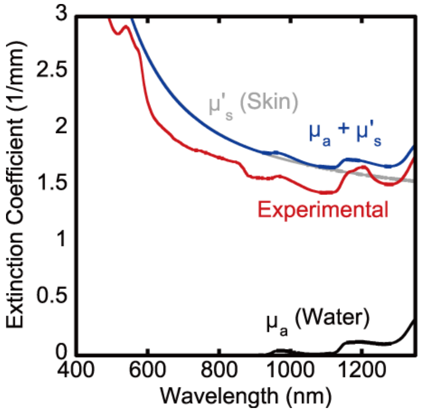

Supplementary Figure 11. Extinction coefficient spectra of mouse skin

The spectra were experimentally measured (red), and estimated from the sum (blue) of the absorption coefficient μ_a of water (black) and the reduced scattering coefficient μ'_s of the skin (gray) based on previously reported scattering properties[1]. In both spectra, the extinction minimum of the mouse skin is in the 1100 nm region.

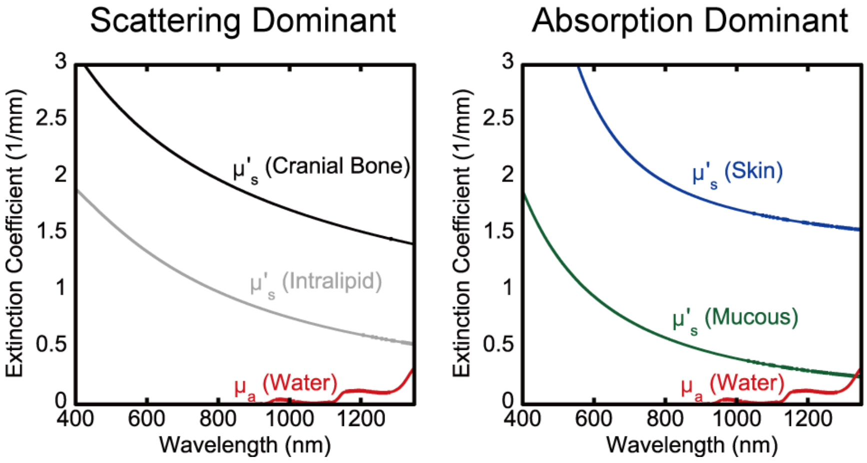

Supplementary Figure 12. Extinction coefficient spectra of biological tissues and Intralipid solution

These spectra are estimated from the reduced scattering coefficients μ'_s based on the previously reported scattering properties[1-3]. For comparison, an absorption coefficient μ_a of water is also shown. The scattering properties of cranial bone and Intralipid **(Kabivitrum Inc.)** (left of panel) have a stronger wavelength dependence compared to those of skin and mucous tissue (right of panel) in the 1100 nm region. A simple estimation based on the μ'_s of these tissues and the μ_a of water indicates that the extinction minimum of cranial bone and Intralipid is in the 1300 nm region (scattering dominant), while that of skin and mucous tissues is in the 1100 nm region (absorption dominant).

Supplementary Methods

Supplementary Method 1, Single-chirality separation of other species

For the separation of other species, multicolumn separation was performed using three columns equilibrated with different SDS concentrations. Approximately 3 mL of allyl dextran-based gel beads (Sephacryl S-200 HR, GE Healthcare) was packed in plastic medical syringes equipped with a cotton filter on the outlet of syringes. The absorption procedure was divided into three steps. For the first step, approximately 1.8 mL of SWCNT dispersion with 0.5% SC and 2.0% SDS was loaded into the first column (all surfactant concentrations were weight percent). After the elution and collection of unbound SWCNTs, the filtrate was diluted to obtain SC and SDS concentrations of 0.5 and 1.0%, respectively. In the same manner, the second (0.5% SC and 1.0% SDS) and

third steps (0.5% SC and 0.5% SDS) were performed. The SWCNTs that adsorbed to each column were eluted and collected by adding DOC and increasing the concentration as follows. For the first column, the DOC concentration was increased from 0.04 to 0.4% in 0.04% steps with fixed concentrations of 0.5% SC and 2.0% SDS. (7,3) SWCNTs were obtained at 0.16% DOC. For the second column, the DOC concentration was increased from 0.02 to 0.2% in 0.02% steps with fixed concentrations of 0.5% SC and 1.0% SDS. (9,4) and (10,3) SWCNTs were obtained at 0.16 and 0.18% DOC, respectively. For the third column, the DOC concentration was increased from 0.01 to 0.1% in 0.01% steps with fixed concentrations of 0.5% SC and 0.5% SDS. (6,4), (6,5), and (8,6) SWCNTs were obtained at 0.02, 0.03, and 0.08% DOC, respectively. For (10,2) SWCNTs, high-purity separation requires a more detailed stepwise elution because of the many competitive species in the SC/SDS/DOC system. The precise stepwise elution was performed using a chromatography system (AKTAexplorer 10S, GE Healthcare). Approximately 2.4 mL of allyl dextran-based gel beads (Sephacryl S-200 HR, GE Healthcare) was packed in a column (Tricorn 10/20 column, GE Healthcare). After equilibration with a 1.0% SDS and 0.5% SC solution, 0.5 mL of the SWCNT dispersion with 1.0% SDS and 0.5% SC, followed by the 1.0% SDS and 0.5% SC solution, was loaded into the column at a flow rate of 1 mL/min. The adsorbed SWCNTs were eluted and collected by adding DOC and increasing the concentration from 0.01 to 0.2% in 0.005% steps with fixed concentrations of 0.5% SC and 1.0% SDS. (10,2) SWCNTs were obtained at 0.08% DOC.

Supplementary References

1. Bashkatov, A. N., Genina, E. A., Kochubey, V. I., Tuchin, V. V. Optical properties of human skin, subcutaneous and mucous tissues in the wavelength range from 400 to 2000 nm. *J. Phys. D Appl. Phys.* **38**, 2543-2555 (2005).

2. Bashkatov, A. N., Genina, E. A., Kochubey, V. I., Tuchin, V. V. Optical properties of human cranial bone in the spectral range from 800 to 2000 nm. *Proc. SPIE* **6163**, 16310 (2006).

3. Vanstaveren, H. J., Moes, C. J. M., Vanmarle, J., Prahl, S. A., Vangemert, M. J. C. Light-scattering in intralipid-10-percent in the wavelength range of 400-1100 nm. *Appl. Optics* **30**, 4507-4514 (1991).
